# An efficient chromatin immunoprecipitation (ChIP) protocol for studying histone modifications in peach reproductive tissues

**DOI:** 10.1186/s13007-022-00876-0

**Published:** 2022-03-31

**Authors:** Monica Canton, Silvia Farinati, Cristian Forestan, Justin Joseph, Claudio Bonghi, Serena Varotto

**Affiliations:** 1 Department of Agronomy, Food, Natural resources, Animals and Environment (DAFNAE), University of Padova, Legnaro, PD Italy; 2 Department of Agricultural and Food Sciences (DISTAL), University of Bologna, Bologna, Italy

**Keywords:** ChIP assay, Peach, Floral buds, Fruit, H3K4me3, H3K27me3

## Abstract

**Background:**

Perennial fruit trees display a growth behaviour characterized by annual cycling between growth and dormancy, with complex physiological features. Rosaceae fruit trees represent excellent models for studying not only the fruit growth/patterning but also the progression of the reproductive cycle depending upon the impact of climate conditions. Additionally, current developments in high‐throughput technologies have impacted Rosaceae tree research while investigating genome structure and function as well as (epi)genetic mechanisms involved in important developmental and environmental response processes during fruit tree growth. Among epigenetic mechanisms, chromatin remodelling mediated by histone modifications and other chromatin-related processes play a crucial role in gene modulation, controlling gene expression. Chromatin immunoprecipitation is an effective technique to investigate chromatin dynamics in plants. This technique is generally applied for studies on chromatin states and enrichment of post-transcriptional modifications (PTMs) in histone proteins.

**Results:**

Peach is considered a model organism among climacteric fruits in the Rosaceae family for studies on bud formation, dormancy, and organ differentiation. In our work, we have primarily established specific protocols for chromatin extraction and immunoprecipitation in reproductive tissues of peach (*Prunus persica*). Subsequently, we focused our investigations on the role of two chromatin marks, namely the trimethylation of histone H3 at lysine in position 4 (H3K4me3) and trimethylation of histone H3 at lysine 27 (H3K27me3) in modulating specific gene expression. Bud dormancy and fruit growth were investigated in a nectarine genotype called Fantasia as our model system.

**Conclusions:**

We present general strategies to optimize ChIP protocols for buds and mesocarp tissues of peach and analyze the correlation between gene expression and chromatin mark enrichment/depletion. The procedures proposed may be useful to evaluate any involvement of histone modifications in the regulation of gene expression during bud dormancy progression and core ripening in fruits.

**Supplementary Information:**

The online version contains supplementary material available at 10.1186/s13007-022-00876-0.

## Background

The edible Rosaceae crops display an extraordinary spectrum of fruit types, including fleshy peach, apple, and strawberry, that provide unique contributions to a healthy diet for consumers forming an important source of an array of secondary metabolites with tangible roles in reducing risks for different diseases [1]. Amongst them, Rosaceae fruit trees represent excellent models for studying not only fruit growth/patterning but also the progression of the reproductive cycle that depends upon environmental conditions. In fact, in a majority of temperate or boreal species, the reproductive cycle, from flower bud initiation to fruit/seed maturation, is completed in 2 years after experiencing cold temperature to overcome a winter rest period, called dormancy. To date, the reproductive cycle has been largely investigated both in physiological and molecular terms, but not in a comprehensive manner. As an initial broad observation, these studies established that the reproductive cycle resulted from coordinated changes in expression of hundreds to thousands of genes involved in the regulation of structural (cells/tissues differentiation of flower and fruit) and metabolic (fruit metabolites and hormones) traits of reproductive organs [2, 3]. However, the attention has mainly been paid to the fruit and its ripening phase: in literature, there is an impressive number of studies focused on understanding the main processes that take place during the progression from an immature to ripe fruit, whereas the earlier phases, corresponding to the pre-pollination stage are not well clarified [4]. In particular, the comprehension of the bud dormancy phase is strategic when considering the impact of climate change on plant physiology. Dormancy is an evolutionary process that is entrusted to temperature resilient structures such as buds and can be interpreted as “a state of self-arrest of the shoot apical meristem (SAM) which is maintained under growth-promoting conditions” [5, 6]. Therefore, dormancy mainly ensures survival under extremely low temperatures during winter and can also influence developmental functions, including fruit setting and patterning, when inadequate temperature compromises overcoming dormancy [7].

The emerging progress of high-throughput methods and bioinformatics technologies for analysing genome structure and function has had an important impact on research in fruit trees and has significantly contributed towards accelerating the discovery of specific DNA regulatory elements that interact with transcription factors (TFs) responsible for plant growth and responses to plant–environment interaction [8]. For instance, whole-genome sequencing projects of two fruit crop species, namely peach and apple, have fostered in-depth molecular studies in *Prunus* and *Malus* species over recent years [9–11], allowing the identification of factors that interact in a multilevel process and triggers the coordinated action of master regulators, including hormone signalling, microRNAs, and epigenetic mechanisms [12]. Among the latter, chromatin-remodelling mechanisms, mediated by both histone modifications and other chromatin-related processes, play a crucial role in gene modulation, by influencing the ability of transcription factors to bind DNA regulatory elements and thereby controlling gene expression [13]. However, the vast majority of plant cis-elements in gene promoters are unknown [14], because optimised experimental protocols for recovering nucleic acids to be utilized for genome-wide analyses are still lacking.

A very useful technique to investigate DNA–protein interaction and chromatin states and their dynamics is chromatin immunoprecipitation (ChIP). It relies on the use of a specific antibody raised against a target transcription factor or the histone modification under investigation and it is widely used for a few model systems, including Arabidopsis, though it is still remarkably challenging to implement in other plant systems [15, 16]. In plants, ChIP is generally applied for studies on chromatin states and enrichment of post-transcriptional modifications (PTMs) in histone during development or in regulating gene expression during stress response [17]. The integration of expression data, at either single gene or genome-wide level, with histone PTM enrichments in specific gene contexts can unveil possible direct correlations between gene transcriptional variations and histone modification dynamics, during plant tissue differentiation and development. Recent studies on fruit tissues revealed that a native chromatin immunoprecipitation protocol (N-ChIP), performed without cross-linking, is better for profiling histones and histone modification studies for improved antibody specificity, higher pull-down efficiency, lower background, and less bias when compared to an X-ChIP (cross-linked chromatin followed by immunoprecipitation) procedure [18]. In this work, we describe a protocol for ChIP procedure in different reproductive tissues (flower buds and fruit) of *Prunus persica* to investigate histone mark distributions*.* Interestingly, histone modifications have been implicated in regulating both bud dormancy and fruit development progression [19, 20] and peach is claimed as a model in the Rosaceae family for studies of biological processes like bud formation/dormancy and differentiation/development of climacteric fruits [10]. Here we describe a method for chromatin extraction and immunoprecipitation to optimize an X-ChIP protocol, suitable for subsequent gene target (ChIP-qPCR) and genome-wide analyses (ChIP-seq). We aim to figure out a possible correlation between gene expression and the presence of specific chromatin marks. We focused our investigations on the role of two chromatin marks (described in detail in “Results” section), namely trimethylation of histone H3 at lysine in position 4 (H3K4me3) and trimethylation of histone H3 at lysine 27(H3K27me3), in modulating specific gene expression during these fundamental biological processes in the nectarine cultivar Fantasia.

## Results and discussion

### Assessment of chromatin quality: efficiency check of chromatin fixation/extraction and fragmentation

We have developed and optimized an immunoprecipitation protocol on crosslinked chromatin (X-ChIP) suitable for analysing either genome-wide distributions and/or specific distribution at target loci of single post-transcriptional histone modifications in peach reproductive tissues. One of the main advantages of the procedure proposed here is its applicability to hard plant tissues (i.e. Flower buds; FB and fruits mesocarp; FM) stored at − 80 °C after freezing in liquid nitrogen.

Previous studies have described how the success of a ChIP procedure depends upon both the nature of the starting material and the initial processing steps. In these studies, the use of fresh and unfrozen plant tissue as starting material for chromatin extraction and subsequent analyses have often been strongly recommended [21]. Moreover, the use of vacuum-mediated infiltration has also been suggested to ensure an efficient penetration of the fixative for the crosslinking reaction into the plant tissue, which is the crucial step that distinguishes an N-ChIP from X-ChIP procedure [16, 18]. However, in both methods preserving chromatin structure during the isolation and subsequent steps is the main aim of the procedure. For this reason, the cross-reaction with the fixation agent (in our case formaldehyde) in an X-ChIP strategy was the initial step to be evaluated and optimized in our experiments. Insufficient crosslinking will not preserve the chromatin structure, while over crosslinking will hamper the ChIP procedure as reported by [22].

**Fig. 1:**
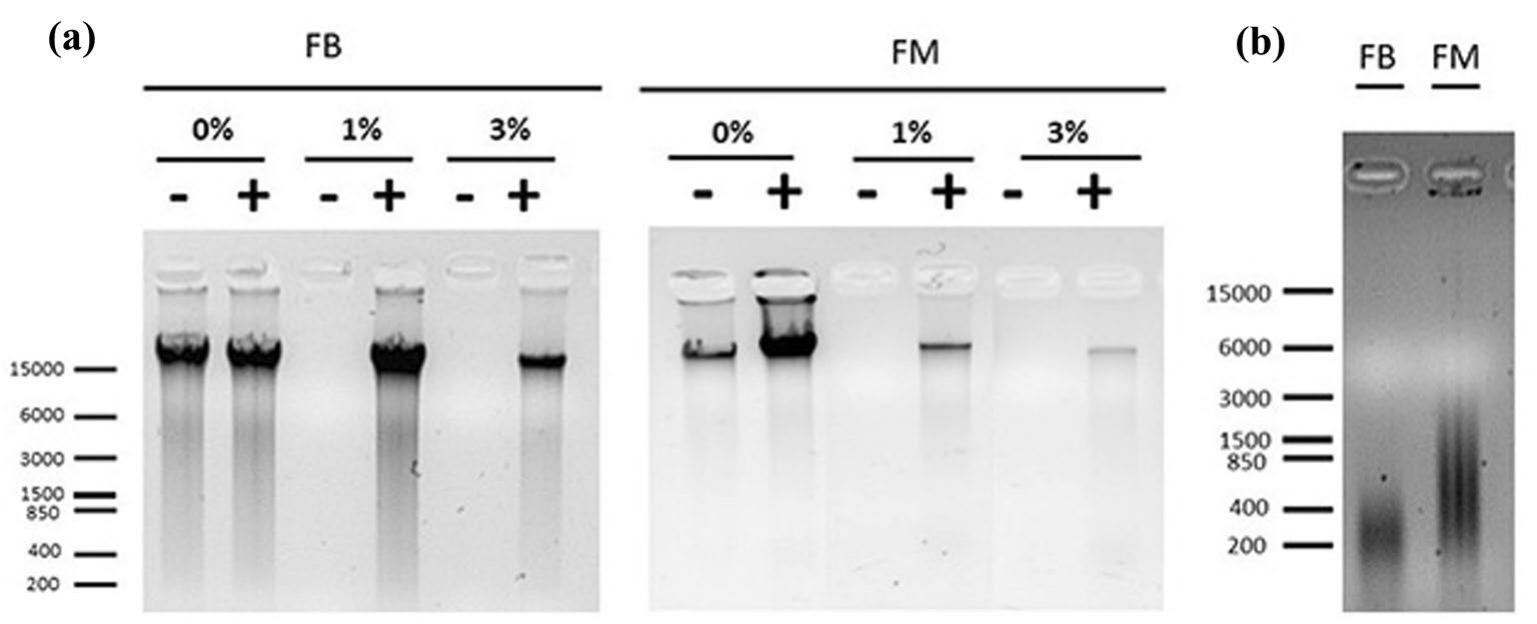
Crosslinking efficiency and physical shearing chromatin analyses. **a** Bud and mesocarp tissues were crosslinked in buffers containing increasing amounts of formaldehyde (0, 1, and 3%). Samples were subjected or not to a reverse crosslinking phase (decrosslinked sample + and −, respectively), and DNA was isolated using phenol/chloroform extraction as described in “Materials and methods” section. While DNA is efficiently isolated from samples that were not crosslinked (lanes indicated with 0%), a decrosslinking procedure is required for the isolation of DNA from cross-linked samples (with 1% indicating the relative concentration of formaldehyde used in testing analyses, which resulted in a better yield of signal). **b** Chromatin shearing check after the application of 60% amplitude with several 10 s shearing rounds (25 for FB and 15 for FM) followed by a reverse crosslinking phase and a DNA isolated using phenol/chloroform extraction

In Fig. 1A, the efficiency estimation conducted to determine the optimal crosslinking conditions for FB and FM tissues is reported. In FB, the developed protocol for chromatin fixation and extraction was very efficient in all three stages (0, 475, and 770CU) during endodormancy. With the progression of dormancy buds have an increase in the number of scales and in the accumulation of starch, which hinders chromatin extraction. Bud scales act as protection for newly formed leaves and branch outgrowth. They form at the end of the growing season once the leaves have fallen off the branches. Despite the absence of scale removal from buds in other published protocols [16], we have proceeded with their removal obtaining a high yield of chromatin in all sample extractions (Fig. 1A). Furthermore, this preventive step allowed us to shorten the number of chromatin extraction phases, avoiding the use of β-mercaptoethanol in our experiments.

For FM, the efficiency of chromatin fixation and the following extraction steps depended on the fruit developmental stage. In the fruit pre-ripening phase, from the onset to the end of the S3 phase, the chromatin extraction was reasonable: the extraction was satisfactory in FM collected at 83, 104, and 111 days after full bloom (DAFB) when the fruit expansion and endoreduplication processes occur [20]. Indeed, during these developmental stages, the accumulation of sugars and other metabolites is reduced due to the high energy requirement for the expansion of FM cells [23]. On the contrary, in samples collected at 118 and 125 DAFB, the high level of polysaccharides and other secondary metabolites caused an inhibitory effect on the extraction phase, resulting in a dramatic reduction of quality and yield (data not shown), as also reported in other works [19, 24, 25]. However, considering both the cellular uniformity of the mesocarp tissue and the general technical limits of the procedure, the chromatin extraction procedure was performed after pooling three biological replicates with the results reported in the following sections.

According to other works conducted on different plant tissues [22], our results with FB and FM indicated that the addition of formaldehyde 1% (v/v) into the fixation starting buffer is more efficient for the following ChIP steps in comparison to the addition of 3% of formaldehyde (v/v), since the former allows recovery of a substantial amount of DNA from the reverse crosslinking step meanwhile the chromatin is neither over- or under-crosslinked (Fig. 1A).

The size of chromatin fragments used as input material is the second determinant factor for the resolution and success of a ChIP procedure since a proper size distribution of DNA fragments is crucial for specific ChIP applications. Ideally, the bulk of the chromatin for the following application includes a length between 250 and 750 bp but, depending on the intended use of ChIP applications (gene target vs the whole genome sequencing), an appropriate shearing step must be determined for each chromatin preparation. In Fig. 1B we report the best results obtained after testing different sonication conditions, in terms of number, timing, and amplitude of rounds. We decided to use a sonication procedure vs an enzymatic MNase-mediated fragmentation since formaldehyde crosslinking restricts access to chromatin for the enzyme [26]. After testing various sonication conditions, we used 60% of amplitude with 15 (for FM) and 25 (for FB) rounds of sonication of 10 s duration. These conditions yielded a physical shearing of chromatin compatible with the following purposes. For our samples, a smear between 200 and 500 bp was observed for FB and between 200 and 800 bp for FM (Fig. 1B). In both cases, the fragmentation was suitable for the following molecular analyses and, in particular, the higher fragmentation of chromatin obtained from buds allowed for successful library preparation for ChIP-Seq analysis accordingly [16].

### Assessment of chromatin immunoprecipitation assay by QPCR and ChIP sequencing in reproductive peach tissues

After the application of specific precautions in the chromatin fixation/extraction protocol, the quality and the pull-down efficiency of the immunoprecipitation were tested. The aim was to define whether our operative changes specific for each peach plant material, affected the results of the following molecular procedures to be performed.

To monitor chromatin states and find out potential correlations between gene transcript levels and enrichment in specific histone modification, two euchromatin regulative histone marks of interest were examined: trimethylation of lysine in position 4 of histone H3 (H3K4me3), which represents an active mark typically enriched around TSS of transcribed genes, and H3K27me3, a silencing mark generally distributed over the whole gene coding region [27–29].

**Fig. 2:**
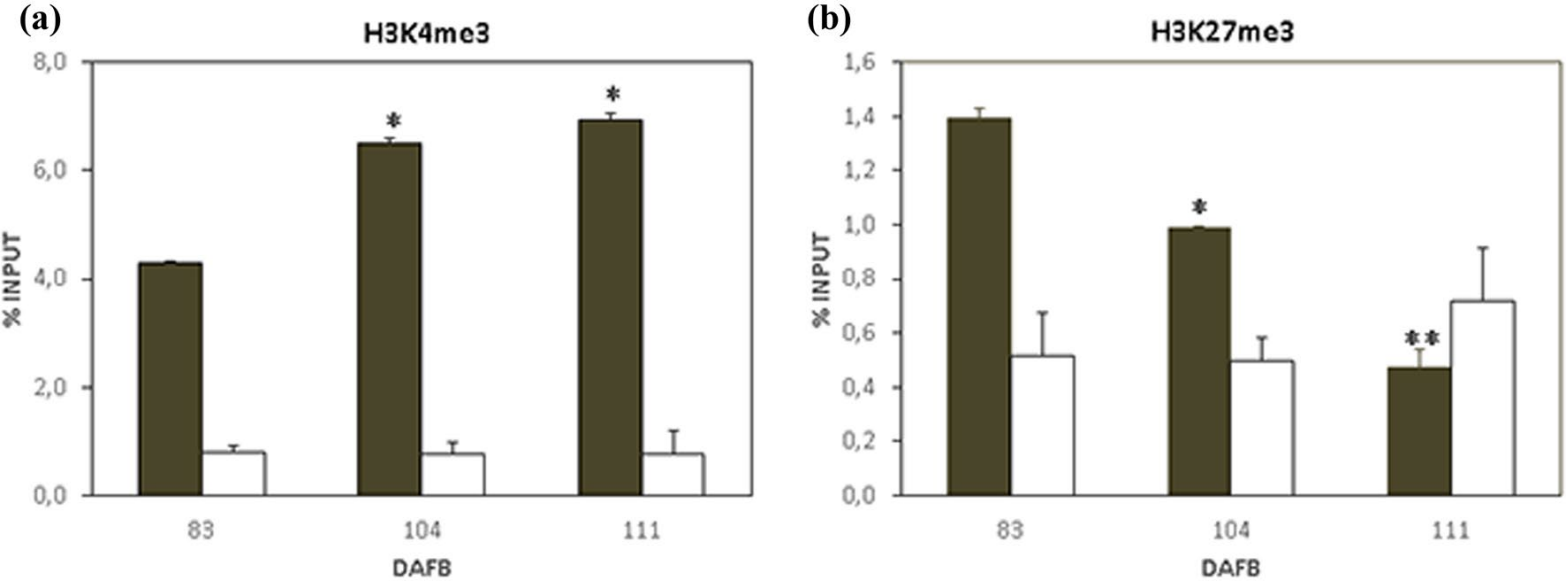
Chromatin marks analysis by X-ChIP method for peach mesocarp tissue. Histone modification analysis on chromatin extracted from FAN mesocarp tissue at 83, 104, and 111 DAFB. The ‘TSS around-’ (dark bars, questioned with primer set A) and ‘gene body-’ (white bars, questioned with primer set C) subregions of *FLESHY* genomic locus, were investigated by real-time PCR quantification on ChIPed DNA immunoprecipitated with α-H3K4me3 (**a**), and α-H3K27me3 (**b**). Data are reported as a percentage of chromatin input (% IP), normalized on background signal (No Ab serum control sample, measured by omitting antibody during ChIP procedure). Three PCR repetitions for each ChIP assay. Standard errors are reported. Asterisks indicate statistically significant changes: * = p ˂ 0.05, ** = p ˂ 0.01. *DAFB* Days after full bloom

For the mesocarp tissue, as target gene, we focused the attention on *PpFLESHY* (PRUPE_6G159200)*,* also known as HECATE3 (HEC3)-like, a TF with a putative key role in fleshy fruit mesocarp tissue identity [30]. In the FAN fruits, *PpFLESHY* did not exhibit relevant variations in its expression level during S1 and S2, while its transcript level increased at S3 and highly accumulated at early S4 [30]. This expression pattern was reconfirmed by our investigations on *FLESHY* transcript levels during different fruit developmental phases (Additional file 1: Fig. S1, Additional file 5). After chromatin immunoprecipitation with the two specific Abs against H3K4me3 and H3K27me3 modified histones, qPCR assays were performed to verify and semi-quantify the presence of *FLESHY* in ChIPed DNA populations, following the indication reported by Rossi et al. [21] (Fig. 2). Based on the different typical distribution patterns of H3K4me3 and H3K27me3 histone marks along the gene sequences, we analysed the *FLESHY* sequence by considering its predicted ‘TSS around-’ and ‘gene body-’ subregions using four couples of primers (A, B couples for ‘TSS around-’ and C, D couples for ‘gene body-’ subregions. For details see Additional file 4: Table S1). qPCR results demonstrated significant enrichment of H3K4me3 activation mark at the level of *FLESHY* ‘TSS around-’, in comparison to that measured in the ‘gene body-’subregion (dark *vs* white bars). Additionally, while no enrichment was observed at the ‘gene body’ during 83, 104, and 111 DAFB, a higher and significant increase (relative to the input), ranging from 4.3 up to 7%, was measured at the ‘TSS around’ region (Fig. 2A). The H3K4me3 preferential enrichment at the TSS level is in agreement with data reported in the literature in other species and tissues [29, 31, 32].

An opposite trend was observed for the silencing mark H3K27me3: a lower enrichment, in terms of % IP of this mark, in all samples in comparison to that observed for the activating mark H3K4me3 in both the investigated gene regions. However, at the TSS level a small reduction of H3K27me3 was measured during the progression of fruit growth, with values ranging from 1.4 to 0.5%, while at gene body level a comparatively lower and constant signal was measured with a less pronounced fold change (Fig. 2B). To exclude reduced Ab efficiency, we analysed in the same mesocarp samples, the putative enrichment of H3K27me3 into a Polygalacturonase family member (*ppePG22*, PRUPE_4G262200), known to be a representative silenced locus during the same developmental stages (Additional file 2: Fig. S2). The analysis confirmed the enrichment of this histone mark along the whole gene sequence.

In parallel, we investigated the enrichment of histone modification H3K4me3 and H3K27me3 in FB tissues by performing chromatin immunoprecipitation followed by Illumina sequencing (ChIP-Seq).

**Fig. 3:**
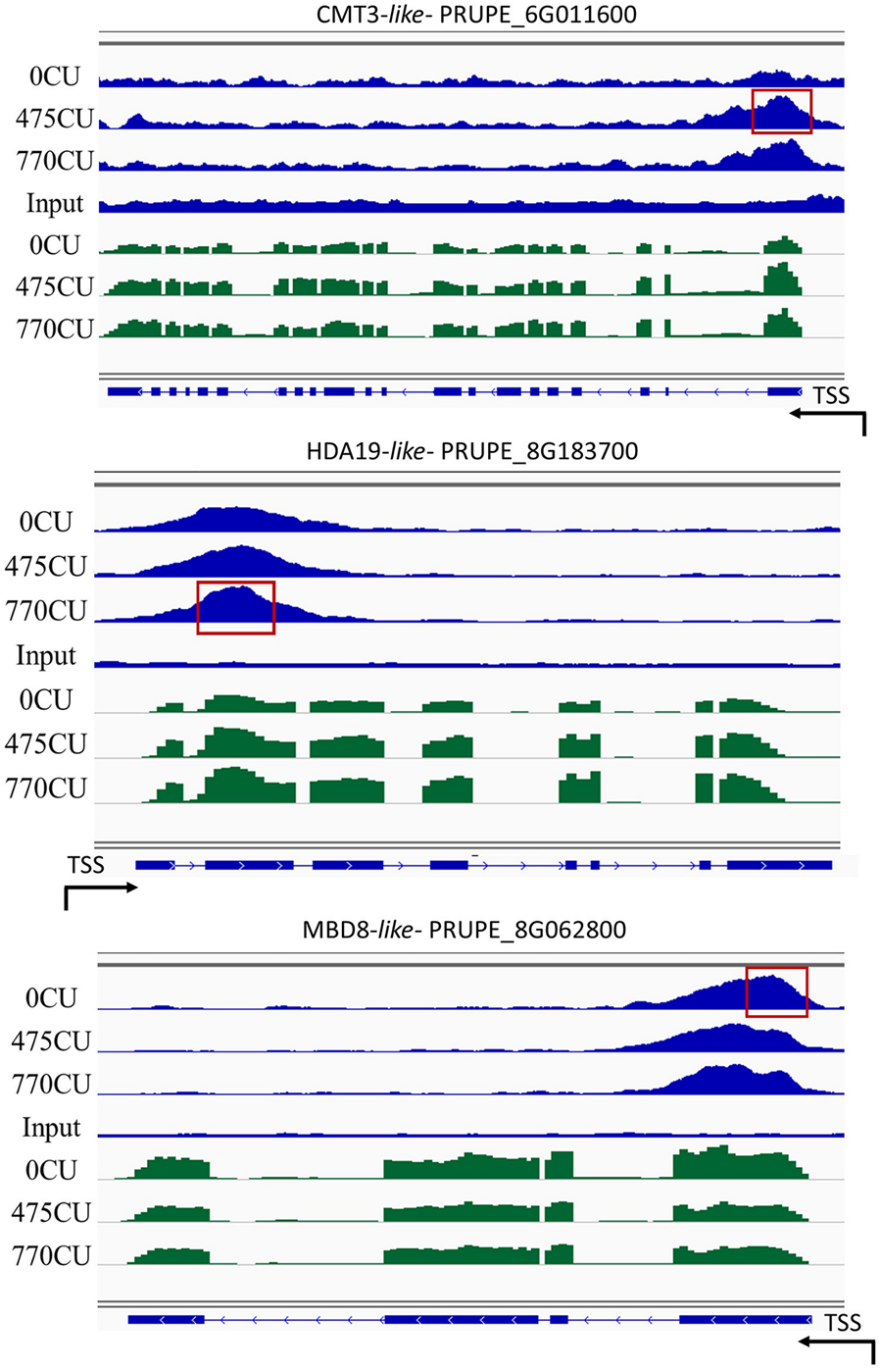
H3K4me3 distribution on selected target genes and their relative expression levels in dormant floral buds. Integrative Genomics Viewer (IGV) screenshot for H3K4me3 signals across PRUPE_6G011600, PRUPE_8G183700, and PRUPE_8G062800 genes compared with those of RNA-Seq (green reads) performed in dormant flower buds collected at 0, 475, and 770CU along with their corresponding inputs. Gene structures are represented by blue rectangles. Arrows represent the TSS, Transcription Start Site, and indicate the gene orientation on the genome. Red boxes represent the differentially expressed peaks

**Fig. 4:**
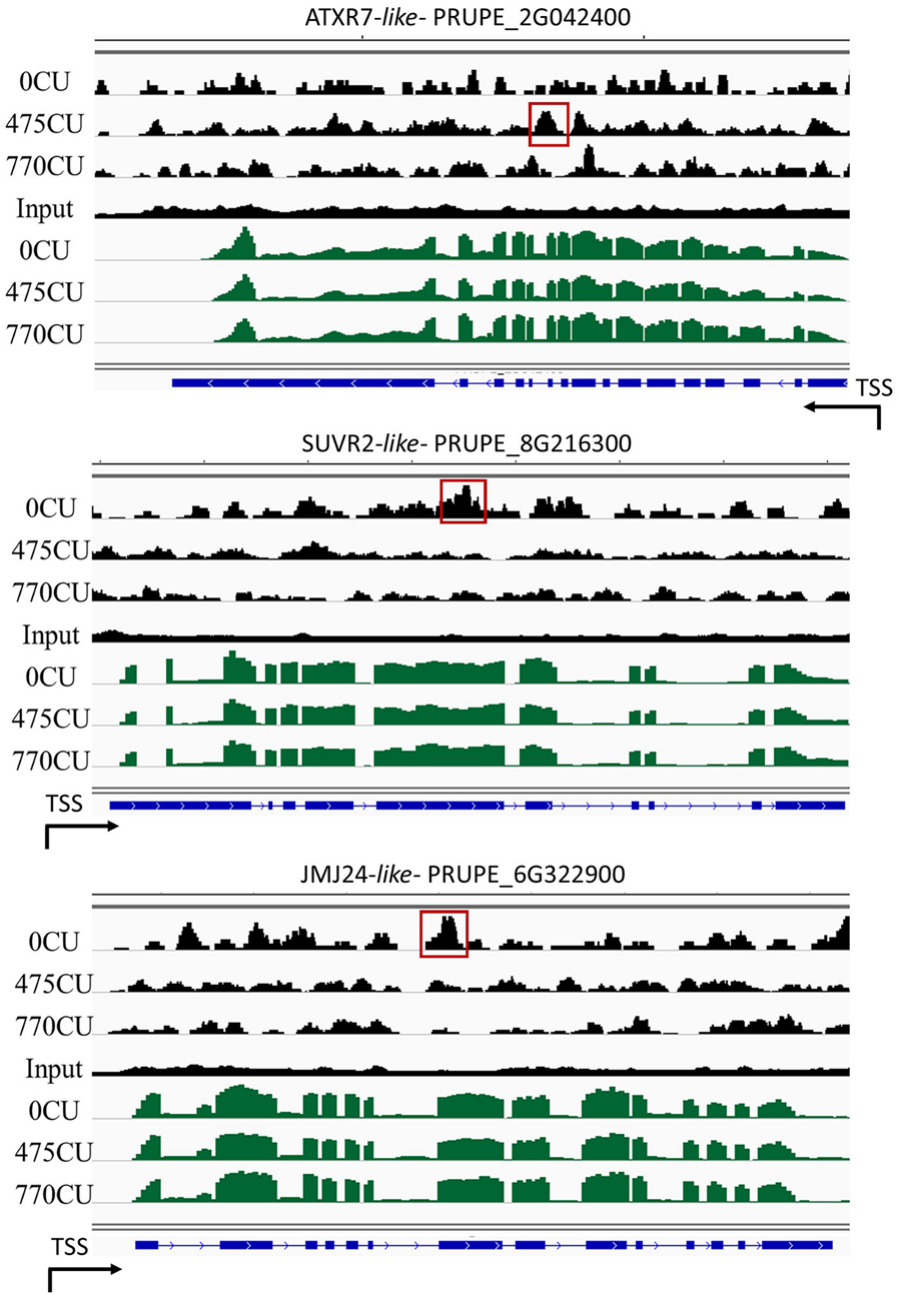
H3K27me3 distribution on selected target genes and their relative expression levels in dormant floral buds. Integrative Genomics Viewer (IGV) screenshot for H3K27me3 signals across PRUPE_2G042400, PRUPE_8G216300, and PRUPE_6G322900 genes compared with those of RNA-Seq (green reads) performed in dormant flower buds collected at 0, 475, and 770CU along with their corresponding inputs. Gene structures are represented by blue rectangles. Arrows represent the TSS, Transcription Start Site, and indicate the gene orientation on the genome. Red boxes represent the differentially expressed peaks

Subsequently, combined biological replicates were loaded into the Integrative Genomics Viewer (IGV) genome browser to visualize both H3K4me3 and H3K27me3 peaks simultaneously with gene expression (RNA-Seq) peaks in the peach genome (Figs. 3 and 4). In Figs. 3 and 4 we report the expression profile of some epigenetic regulator genes identified as marker genes [3] associated with their histone modification profile during endodormancy and endo-ecodormancy transition.

As far as it concerns genes enriched in H3K4me3 (Fig. 3), during the progression of dormancy, active transcription of the CHROMOMETHYLASE 3 (CMT3-like, PRUPE_6G011600) and HDA19-like gene (PRUPE_8G183700) was observed. The DNA methyltransferase CMT3 maintains CHG (H = A, C, or T) methylation at constitutive heterochromatin in plants, and thus it is important for maintaining genome stability [33]. Histone deacetylase 1of Arabidopsis (AtHD1 or AtHDA19), an ortholog of yeast RPD3, is known to be a global regulator of many physiological and developmental processes [34].

A correlation between gene expression and H3K4me3 mark deposition was observed also for a Methyl-CPG-binding domain-like gene (MBD8-like, PRUPE_8G062800). During bud dormancy, for this gene, a depletion of H3K4me3 is associated with a decrease in MBD8-like transcript accumulation. MBDs are proteins with a putative Methyl-CpG-Binding that are considered to function as interpreters of DNA methylation signals [35].

The expression pattern and H3K4me3 distribution at the loci of the reported genes indicate that bud dormancy overcoming is accompanied by the chromatin control of transcriptionally active regions through the enrichment in the H3K4me3 mark.

Regarding H3K27me3, our results indicate that there is not a clear correlation between gene expression and chromatin enrichment in H3K27me3 (Fig. 4). Although this histone mark is responsible for PCR2 mediated gene silencing in euchromatic regions, the enrichment in H3K27me3 does not always correlate with gene silencing or with a low gene expression. This is also the case of some selected chromatin modifiers, such as ATXR7-like (PRUPE_2G042400 a putative ARABIDOPSIS TRITHORAX-RELATED7 a putative Set1 class H3K4 methylase) [36], SUVR2-like (PRUPE_8G216300 a putative SU(VAR)3-9-like histone methyltransferase) [37] and JMJ24-like (PRUPE_6G322900 a jmjC histone demethylase possibly involved in gene silencing) [38]. All of them present a significant decrease in the H3K27me3 mark in their chromatin, during dormancy progression; however, this does not correlate to their transcript level. In the case of flower bud, this missing correlation might be due to the strong tissue specificity of H3K27me3 chromatin mark [39, 40] while flower buds are composed of different specialized tissues.

## Conclusions

**Fig. 5:**
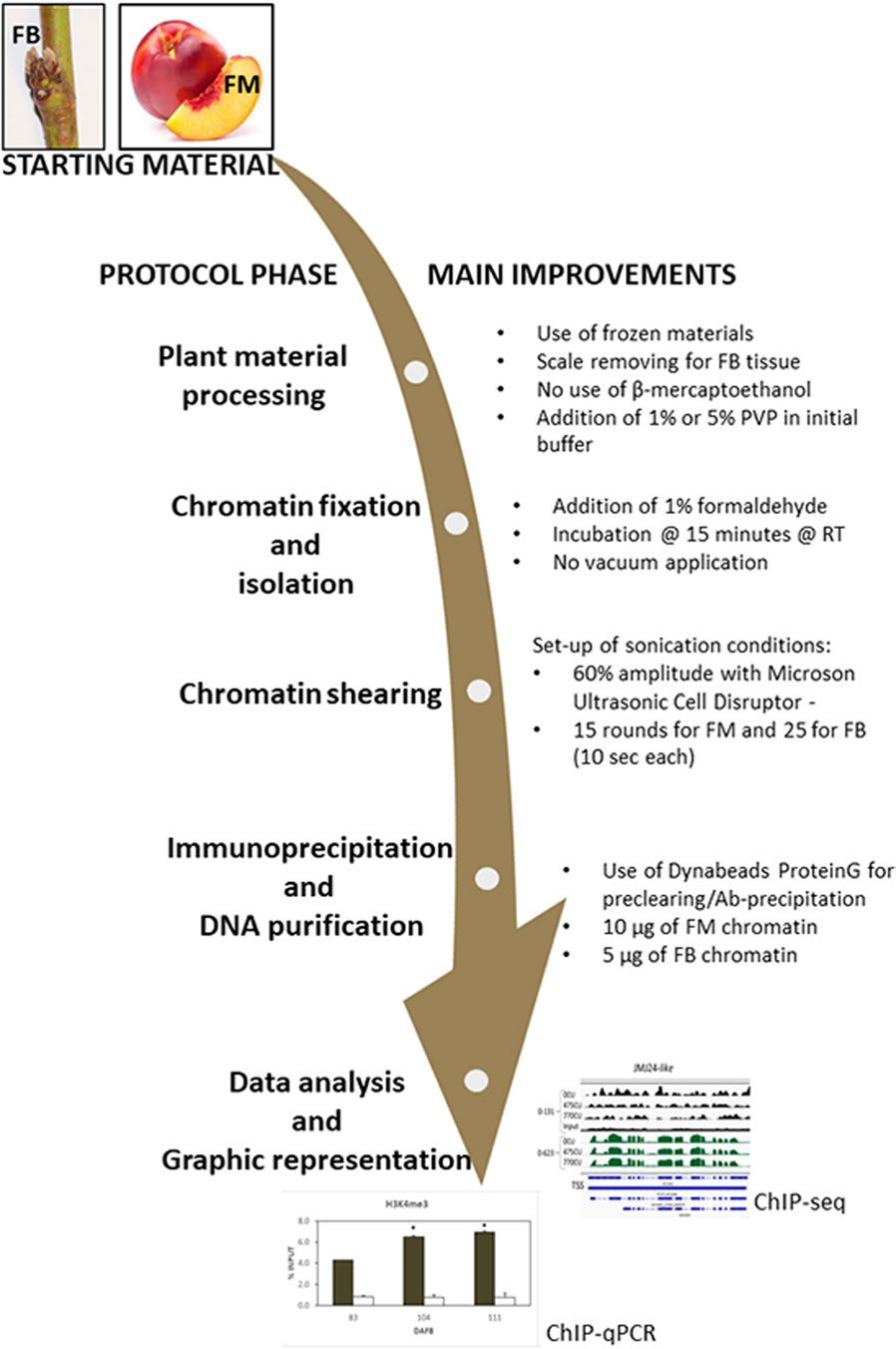
Schematic workflow of ChIP protocol. The protocol phases are reported on the left of the picture and the main relative improvements applied are described on the right

ChIP represents a powerful tool in the study of histone modification dynamics in plant tissues. Several examples in literature strongly support the indispensably important role of the ChIP approach in the field of gene regulation research. The general aim of this report was to describe an optimized X-ChIP procedure for recalcitrant plant tissues because of their structural properties and composition, such as peach reproductive tissues. An affordable and repeatable procedure for studying the distribution of modified histones in buds and fleshy fruits or other plant organs/tissues with high levels of polysaccharides, secondary metabolites (like phenols), high content of water, and large vacuoles has been described in detail (Fig. 5). To summarize, the major advantages of the proposed ChIP protocol, when compared with alternatives already published, are (i) the use of frozen tissues; (ii) avoiding the step for isolation of clean nuclei; (iii) the use of a common NIB extraction buffer for all analysed tissues, and (iv) avoiding the addition of β-mercaptoethanol [16, 18, 41]. Additionally, the preventive elimination of scales guarantees initiation with low amounts of tissue, to get a higher cell/tissue homogeneity and consequently a better biological interpretation of ChIP results.

Furthermore, our procedure was optimized for the following molecular investigations and allowed us to analyse the distribution of histone marks at single-gene level by qPCR or at genome-wide level by NGS sequencing. By integrating the data on gene expression and the enrichment/depletion of specific modified histone at selected loci, we obtained information on the effect of a histone modification on gene expression and correlated the expression change with variations in distribution of chromatin marks as well as enrichment during fruit growth/ripening and bud dormancy in peach.

Results point out a possible involvement of chromatin dynamics in the reproductive cycle of peach, from bud to fruit, similar to what has been observed for other species [12, 42].

## Materials and methods

### Plant material

Ten-year old trees of *Prunus persica* (L. Batsch), cv. Fantasia (FAN), cultivated following the standard horticultural practices, in the experimental farm ‘L. Toniolo’ of the University of Padova, Italy (GPS coordinates: 45° 20′ 48.9″ 110 N 11° 57′ 00.3″ E) were used for bud and fruit samples.

Flower buds (FB) were collected during the winter 2018 and 2019 (05/11/2018, 10/12/2018, 07/01/19), corresponding to 0, 475, and 770 chilling units (CU) respectively, calculated as described by [43]. Daily temperature readings were retrieved from Agenzia Regionale per la Prevenzione e Protezione Ambientale del Veneto (ARPAV; https://www.arpa.veneto.it/). At each time point, buds were collected from groups of 3–4 plants each, corresponding to two (for 0CU and 770CU) and three (for 475CU) biological replicates, their scales were removed and immediately frozen in liquid nitrogen for their storage at − 80° C until subsequent molecular analyses. Fruits were collected following the fruit growth by measuring the equatorial diameter. The fruit double sigmoidal growth kinetics have been divided into four phenological stages, named S1, S2 S3, and S4, as described by [44] (Additional file 3: Fig. S3). Phases corresponding to the first exponential growth phase due to cell division, pit hardening phase, second exponential growth phase mainly owing to cell expansion, and ripening processes respectively, were described in detail in [20]. For conducting the expression analysis, fruit samples collected at 41, 48, 55, 62, 69, 83, 90, 97, 104, 111, 118, 125, and 132 days after full bloom (DAFB) were used. For chromatin extraction and the following immunoprecipitation only samples belonging to S2/S3 transition, S3 and S4 phases (83, 104, 111, and 125 DAFB), were used. A mesocarp portion (FM sample), derived from at least 15 fruits (corresponding to three biological replicates, each composed of five fruits), was collected by taking, from each fruit, a radial section from the epicarp to the first cell layer of the endocarp tissue, with high cellular type homogeneity [20]. This portion was immediately frozen in liquid nitrogen and stored at − 80 °C.

### Reagent setup for chromatin extraction

Nuclear Isolation Buffer (NIB): 10 mM HEPES pH 7.6, 1 M sucrose, 5 mM KCl, 5 mM MgCl_2_, 5 mM EDTA, 0.6% Triton X-100, 0.5 mM PMSF, 1% PVP (**or 5% PVP for mesocarp tissue**).

Percoll solution: 15% Percoll, 10 mM HEPES pH 7.6, 1 M sucrose, 5 mM KCl, 5 mM MgCl_2_, 5 mM EDTA, 5% PVP.

Wash Buffer_FM (Fruit Mesocarp): 10 mM HEPES pH 7.6, 1 M sucrose, 5 mM KCl, 5 mM MgCl_2_, 5 mM EDTA, 5% PVP.

Wash Buffer 1_FB (Floral Buds): 10 mM HEPES pH 7.6, 1 M sucrose, 5 mM KCl, 5 mM MgCl_2_, 5 mM EDTA, 1% PVP.

Wash Buffer 2_FB: 10 mM HEPES pH 7.6, 1 M sucrose, 5 mM KCl, 5 mM MgCl_2_, 5 mM EDTA.

Lysis Buffer: 50 mM HEPES pH 7.6, 150 mM NaCl, 1 mM EDTA, 1% Triton X-100, 0.1% deoxycholate (Sigma, D6750), 0.1% SDS, 10 mM Na-butyrate, and protease inhibitor cocktail (Sigma).

### Reagent setup for chromatin immunoprecipitation

Low Salt Buffer: 20 mM Tris–HCl pH 8.0, 150 mM NaCl, 1% Triton X-100, 2 mM EDTA, 0.1% SDS.

High Salt Buffer: 20 mM Tris–HCl pH 8.0, 500 mM NaCl, 1% Triton X-100, 2 mM EDTA, 0.1% SDS.

LNDET Buffer: 20 mM Tris–HCl pH 8.0, 250 mM LiCl, 1% Nonidet P-40, 1% deoxycholate.

TE Buffer: 10 mM TRIS pH 8.0, 1 mM EDTA.

TE Buffer for Proteinase K: 40 mM TRIS pH 8.0, 10 mM EDTA.

### Chromatin extraction and immunoprecipitation assay

The general procedure described is valid for both FB and FM tissues. All buffers used were prepared using autoclaved stock solutions on the day of use and kept on ice until required. Phenylmethylsulfonyl fluoride (PMSF, Sigma), Na-butyrate and protease inhibitor cocktail (PI, Sigma) should be added into the solutions just before use. The detailed explanations for all steps are reported below, with “**Notes**” describing points specific for each tissue sample.

### Chromatin fixation and isolation

1. Grind the frozen plant tissue to a fine powder with liquid nitrogen in a pre-chilled mortar and pestle. Keep the sample frozen in liquid nitrogen to prevent it from thawing during grinding. Transfer into a pre-chilled 50 ml tube.
2. Suspend the powder in 25 ml of cold NIB and mix well by inverting the tubes until complete homogenization. **Note**: **use the ratio 0.5 g FB or 1.5 g FM tissue in 25 ml NIB**.
3. Fix the chromatin by adding 1% formaldehyde and incubate for 15 min at room temperature (RT) while mixing every 5 min by inverting the tube.
4. Stop the fixation reaction by adding 3.4 ml of 1 M Glycine followed by mixing and inverting the tube for 5 min.
5. Filter the solution through a single layer of Miracloth (Millipore, 475855) into a new 50 ml tube and centrifuge at 5000×*g* for 15 min at 4 °C. Discard the supernatant.
6. Wash the FB pellet with 3 ml of cold Wash Buffer 1_FB and the FM pellet with 5 ml of cold Wash Buffer_FM. Suspend the pellet by mixing the tube.
7. Centrifuge at 5000×*g* for 15 min at 4 °C. Discard the supernatant.
8. Wash the FB pellet with 3 ml of cold Wash Buffer 2_FB and the FM pellet with 5 ml of cold Wash Buffer_FM. Suspend the pellet by mixing the tube.
9. Centrifuge at 5000×*g* for 15 min at 4 °C. Discard the supernatant.
10. **Note Additional step for FM sample. Suspend the pellet in 5 ml of cold Percoll solution. Suspend the pellet by mixing the tube. Centrifuge at 5000×g for 15 min at 4 °C and discard the supernatant**.

### Nuclei lysis and chromatin shearing

11. Suspend the pellet in 500 μl of Lysis Buffer. Store at − 20 °C or proceed with chromatin shearing with the following step 12.
12. Sonicate the chromatin suspension by using Microson Ultrasonic Cell Disruptor XL2005 (Heat Systems, Germany) set at maximum 60% power output to perform 25 (for FB) or 15 (for FM) rounds of sonication of 10 s, followed by 30 s rest in ice, to produce fragments of 200–700 nucleotides. **Note**: **amplitude, time, and the number of pulses should be tested for each chromatin biological sample because different smear signals could be produced.**
  Centrifuge at 16,000×g for 10 min at 4 °C. Recover the supernatant. Save a fraction (500 μl) of the supernatant, to be used with the reverse crosslinking control step (13) for a quality check and the extraction efficiency estimation of the process, and store the remaining sample at – 20 °C until use. The saved supernatant fraction will be use used as an input sample (IP) in the following PCR evaluation.
13. Reverse crosslinking control:
  Sub-divide the saved fraction from the previous step into two 1.5 ml tubes, to have a control de-crossed (+ DC, in which the reverse crosslinking reaction will be carried out) and a control, not de-crossed (− DC, in which the reverse crosslinking reaction will not be carried out).
  Add 0.2 M NaCl in + DC fraction and incubate both + DC and − DC for 16 h at 65 °C.
  Add an equal volume of phenol/chloroform/isoamyl alcohol (25:24:1). Vortex well and centrifuge at 16,000×g for 10 min at RT.
  Recover the supernatant in a new 1.5 ml tube and repeat the step by adding an equal volume of phenol/chloroform/isoamyl alcohol (25:24:1). Recover the supernatant.
  Add 1/125 volumes of glycogen and 2.5 volumes of 100% EtOH to the recovered phase and incubate at − 20 °C for at least 2 h for DNA precipitation.
  Centrifuge at 16,000×g for 30 min at 4 °C.
  Wash the pellet with 500 μl 70% EtOH. Centrifuge at 16,000×g for 5–10 min at 4 °C.
  Dry the pellet at room temperature and add 15–30 μl of double distilled water to resolve the DNA.
  Perform RNase treatment by adding 1 mg/ml of Rnase A and incubating for 30 min at 37 °C.
  Check the efficiency of chromatin fixation/extraction and fragmentation by running on agarose gel 1% comparing the signal visualized in + DC vs − DC samples.

### Chromatin immunoprecipitation by dynabeads™ protein G

#### Day 1

1. Dynabeads Protein G preparation
  Before proceeding with the preparation of Dynabeads™ Protein G aliquots (ThermoFisher Scientific, 10004D), it is necessary to evaluate the number of biological samples and antibodies that will be employed including the negative control that is the sample without antibodies. The following steps are referred to as a single aliquot.
  1.1 Vortex the Dynabeads™ Protein G and pipette 100 μl in an empty 1.5 ml tube.
  1.2 Centrifuge at 700×*g* for 2 min at RT**. Note: Before using the magnetic separator, we suggest the use of a centrifuge to collect at the bottom the beads to facilitate the following separation with a magnetic rack).**
  1.3 Use a magnetic separator rack (ThermoFisher Scientific, 12321D) to remove the supernatant and add 1 ml of Lysis Buffer to the beads.
  1.4 Incubate the tubes at RT on a rotating incubator with gentle rotation for 5 min.
  1.5 Centrifuge 700×*g* for 2 min at RT.
  1.6 Repeat the wash steps (1.3–1.5) three more times.
  1.7 Add 100 μl of Lysis Buffer. Mix well by pipetting.
  1.8 Sub-divide the Dynabeads™ Protein G into two aliquots (50 μl each) and store them on ice.
2. Pre-clearing of chromatin sample
  2.1 Centrifuge one of the tubes containing 50 μl of Dynabeads™ Protein G at 700×*g* for 2 min at RT.
  2.2 Use a magnetic separator rack to remove the supernatant.
  2.3 Transfer chromatin sample previously extracted and sheared, into the tube containing Dynabeads^TM^ Protein G pellet. **Note**: **5 μg chromatin from FB and 10 μg from FM tissue.**
  2.4 Incubate the tubes at 4–10 °C on a rotating incubator with gentle rotation for 4–5 h.
3. Antibodies preparation
  3.1 Prepare 250 μl of Lysis Buffer containing 5–7 μg of antibody against H3K4me3 (Active Motif, 39159) or 10 μg of antibody H3K27me3 (Millipore, 07-449). Preserve on ice until use.
  3.2 Centrifuge at 700×*g* for 2 min at 4 °C the second tube containing 50 μl of Dynabeads™ Protein G previously prepared in step 1.8.
  3.3 Remove the supernatant using a magnetic separator rack and add the 250 μl of Lysis Buffer + Antibody prepared in step 3.1.
  3.4 Incubate the tubes at 4–10 °C on a rotating incubator with gentle rotation for 2 h.
  3.5 Centrifuge at 700×*g* for 2 min at 4 °C.
  3.6 Use a magnetic separator rack to remove the supernatant and add the 500 μl of Lysis Buffer.
  3.7 Incubate the tubes at RT on a rotating incubator with gentle rotation for 5 min.
  3.8 Centrifuge at 700×*g* for 2 min at 4 °C.
  3.9 Use a magnetic separator rack to remove the supernatant and add the 500 μl of Lysis Buffer + BSA 5mg/ml (First Wash).
  3.10 Incubate the tubes at RT on a rotating incubator with gentle rotation for 5 min.
  3.11 Centrifuge at 700×*g* for 2 min at 4 °C.
  3.12 Repeat steps 3.9–3.11 twice.
  3.13 Use a magnetic separator rack to remove the supernatant and add the 500 μl of Lysis Buffer containing BSA 5 mg/ml. Incubate the tubes at 4–10 °C on a rotating incubator with gentle rotation for 2 h.
4. Immunoprecipitation
  4.1 Centrifuge the Dynabeads Protein G derived from step 3.13 at 700×*g* for 2 min at 4 °C.
  4.2 Use a magnetic separator rack to remove and discard the supernatant. Place the tubes containing the pellet on ice.
  4.3 Centrifuge the tubes containing the chromatin + Dynabeads™ Protein G (from pre-clearing steps) at 700×*g* for 2 min at 4 °C.
  4.4 Use a magnetic separator rack to recover the supernatant and throw away the Dynabeads™ Protein G pellet. Transfer the pre-cleared supernatant into the tubes containing the pellet derived from step 4.2.
  4.5 Incubate the tubes O/N at 4–10 °C on a rotating incubator with gentle rotation.

#### Day 2

1. Washes and DNA elution

1.1 Centrifuge at 700*g* for 2 min at 4 °C.
1.2 Use a magnetic separator rack to remove the supernatant and add the 500 μl of Low Salt Buffer.
1.3 Incubate the tubes at RT on a rotating incubator with gentle rotation for 5 min.
1.4 Centrifuge at 700×*g* for 2 min at 4 °C.
1.5 Use a magnetic separator rack to remove the supernatant and add the 500 μl of High Salt Buffer.
1.6 Incubate the tubes at RT on a rotating incubator with gentle rotation for 5 min.
1.7 Centrifuge at 700×*g* for 2 min at 4 °C.
1.8 Use a magnetic separator rack to remove the supernatant and add the 500 μl of LNDET Buffer.
1.9 Incubate the tubes at RT on a rotating incubator with gentle rotation for 5 min.
1.20 Centrifuge at 700×*g* for 2 min at 4 °C.
1.21 Use a magnetic separator rack to remove the supernatant and add the 500 μl of TE Buffer.
1.22 Repeat the wash with TE buffer twice.
1.23 Centrifuge at 700×*g* for 2 min at 4 °C.
1.24 Use a magnetic separator rack to remove the supernatant and add the 260 μl of NaHCO_3_ 0.1 M and 1% SDS. Vortex and incubate the tubes at 65 °C on a rotating incubator with gentle rotation for 30 min. Vortex every 15 min.
1.25 Centrifuge at 700×*g* for 2 min at RT.
1.26 Use a magnetic separator rack to recover the supernatant and transfer it to a new 1.5 ml tube.
1.27 Repeat the elution by adding another 260 μl of NaHCO_3_ 0.1M and 1% SDS. Vortex and incubate the tubes at 65 °C on a rotating incubator with gentle rotation for 30 min. Vortex every 15 min.
1.28 Centrifuge at 700×*g* for 2 min at RT.
1.29 Use a magnetic separator rack to recover the supernatant and combine it with the first elution, to get a final volume of 500 μl.
1.30 Add a final concentration of 0.2 M NaCl and incubate for 16 h at 65 °C.

#### Day 3

1. DNA precipitation and purification

1.1 Spin down and add 1/125 volumes of glycogen and 2.5 volumes of EtOH 100%. Mix by inverting the tubes and incubating at − 20 °C for 2 h.
1.2 Centrifuge the samples at 14,000×*g* for 15 min at 4 °C. Discard the supernatant.
1.3 Wash the pellet with 1 ml of EtOH 70%. Vortex to detach the pellet.
1.4 Centrifuge the samples at 14,000×*g* for 15 min at 4 °C. Discard the supernatant.
1.5 Dry well the pellet.
1.6 Suspend the pellet by adding 300 μl of TE buffer for proteinase K. Add 30 μl of RNase A 10 mg/ml and incubate for 30 min at 37 °C.
1.7 Add 2 μl of Proteinase K (10 μg/μl) and incubate for 1 h at 42 °C.
1.8 Add an equal volume of phenol/chloroform/isoamyl alcohol (25:24:1), vortex, and centrifuge at 14,000×*g* for 5 min at RT.
1.9 Recover the supernatant in a new 2 ml tube.
1.10 Follow the protocol for Purification by QIAquick PCR purification kit (QIAGEN) with manufactory instructions.
1.11 Elute in 80 μl of Elution Buffer (supplied by Kit).
1.12 One microliter of this ChIPed DNA and an appropriate dilution of input (from 1:60 to 1:100) can be used for qPCR control (“pull-down efficiency evaluation of immunoprecipitation”) before performing analyses.
1.13 Store the ChIPed DNA at – 20 °C.

### Pull-down efficiency evaluation of immunoprecipitation

One microliter of ChIPed DNA and an appropriate dilution of input (from 1:60 to 1:100) were used for the following Real-time qPCR analyses. For every enrichment of histone marks in each target gene investigation, at least two pairs of primers were considered for PCR reactions at specific regions, and the sequences of best working primers (in terms of dimer formation and specificity), with relative positions compared to predict TSS, are reported in Additional file 4: Table S1. qPCR data analyses were performed as reported in Rossi et al. 2007 and significant differences in the level of each analysed histone mark were assessed by Student t-tests. At least three technical replicates were performed for each sample during the same PCR investigation, and a threshold cycle (TC) mean value with a standard error was calculated. A ΔTC value was calculated by subtracting the TC mean value for the samples to be compared. The following fold difference (FD), for a given primer combination, was then determined by raising 2 to the ΔTC power. Data were graphed as % INPUT since standardized to input chromatin, representing a whole chromatin condition specific for each biological sample. A subtraction of background signal, originating from the omission of antibody in the ChIP experiment in the corresponding sample, was determined.

### ChIP-SEQ analysis

A total of 15 ChIP libraries (2 biological replicates × 2 antibodies (Abs) for the sample 0 and 770 CU and 3 biological replicates × 2 Abs for the sample at 475 CU) and one control library (input) representing whole chromatin (WC) was used for the ChIP-Seq assay. Libraries and sequencing were performed by IGA Technology Services (Udine, Italy) according to the standard operation. Ovation^®^ Ultralow V2 DNA-Seq Library Preparation Kit (NuGEN, Redwood City, CA) was used for library preparation following the manufacturer’s instructions. Immunoprecipitated DNA was quantified by Qubit 2.0 Fluorometer (Invitrogen, Carlsbad, CA). Final libraries were checked with both Qubit 2.0 Fluorometer (Invitrogen, Carlsbad, CA) and Agilent Bioanalyzer DNA assay or Caliper (PerkinElmer, Waltham, MA). Libraries were then prepared for sequencing and sequenced on 20 M reads single-end 75 bp mode on NovaSeq6000 (Illumina, San Diego, CA). For the WC control library, 60 M of 75 bp single-end reads were produced.

FastQC (https://www.bioinformatics.babraham.ac.uk/projects/fastqc/) was used to assess the quality of the reads. The CHIP-Seq raw reads were processed for adapter clipping and quality score trimming using Trimmomatic v 0.39 [45]. Clean reads were mapped to the *P. persica* genome v.2.0 [9] obtained from Ensembl (http://plants.ensembl.org/index.html) using bowtie2 [46]. ChIP‐Seq peaks calling and differential analysis between the three analysed samples were performed using Model-based Analysis of ChIP-Seq (MACS) [47].

For each histone modification, the differentially enriched peaks were calculated by comparing the time points in pairs encompassing three different comparisons (0CU vs 475CU, 475CU vs 770CU, and 770CU vs 0CU). The genes nearest to the differentially enriched peaks were identified and annotated using Homer motif analysis software [48]. Differentially expressed genes in FB (DEGs; p adj < 0.05) discovered in a previous RNA-Seq analysis (see “Availability of data and materials” section) were associated with the identified genes that were H3K4me3 and H3K27me3 enriched and the putative function for each gene was deduced from the *Arabidopsis thaliana* homolog. Transcript sequences were scanned by blastx against UniProt/Swiss-Prot and UniProt/TrEMBL to search for homology.

### RNA extraction and gene expression analysis

For FB, total RNA was extracted from 70 to 80 mg of frozen and ground sample using RNeasy Plant Mini kit (Qiagen) with minor modifications: 1.5% PVP-40 was added in the extraction buffer RLT in a total volume of 750 µl instead 450 µl. On the contrary for FM, total RNA extraction was performed as reported in [30]. For both tissue samples, RNA concentration and quality were determined by measuring OD260/230 and OD260/280 ratio on a NanoDrop 2000c spectrophotometer (Thermo Scientific).

For FM samples, cDNA synthesis was performed with the SuperScript III reverse transcriptase kit (Invitrogen), according to manufacturer’s instructions. Quantitative Real-Time PCR expression (qRT-PCR) analysis was performed using a StepOnePlus™ Real-Time PCR System (Applied Biosystems) and the FAST SYBR^®^ GREEN PCR Master Mix (Thermo Fisher Scientific), following manufacturer’s guidelines. Three technical replicates were carried out for each primer combination in each sample and absolute quantification of gene expression (normalized to UBIQUITIN (UBQ-PRUPE_4G204900 transcript quantities) was performed with the StepOne Software 2.3 (Thermo Fisher Scientific). The primers used are listed in Additional file 4: Table S1. For qRT-PCR oligonucleotides were designed respectively on primary transcripts.

## Supplementary Information

**Additional file 1: Fig. S1.** Expression pattern of *Fleshy* (PRUPE_6G159200) gene throughout the whole fruit development in Fantasia genotype. Quantitative real-time qRT-PCRs were performed throughout fruit development in the mesocarp of cv FAN (for details and primers sequences see “Material and methods” section and Additional file 4: Table S1). Developmental phases S1, S2, S3, and S4, related to the typical growth kinetic of the peach fruit, are indicated at the top of each chart. The analysis shows a differential expression pattern during fruit development, displaying an exponential increase in expression levels starting from the end of S2 up to the S4 stage. qRT-PCRs were performed in triplicate on three biological replicates as described by [1] and [2]. Data were acquired, elaborated, and exported with the StepOne Software v2.3 (ThermoFisher, Waltham, MA, USA) and the average value was graphed. MNE: Mean Normalized Expression, DAFB: days after full bloom. Bars represent standard deviation (n = 3).

**Additional file 2: Fig. S2.** Histone modification analysis on gene body-ppePG22 genomic locus. Chromatin marks analysis by the X-ChIP method was performed on chromatin extracted from FAN mesocarp tissue at 83, 104, and 111 DAFB. The ‘gene body-’ (questioned with two pairs of primer set designed on CDS) were investigated by real-time PCR quantification on ChIPed DNA immunoprecipitated with α-H3K4me3 (black bars, not visible) and α-H3K27me3 (white bars). Data are reported as a percentage of chromatin input (% INPUT), normalized on background signal (No Ab serum control sample, measured by omitting antibody during ChIP procedure). Three PCR repetitions for each ChIP assay. Standard errors are reported. Asterisks indicate statistically significant changes of * = P ˂ 0.05. DAFB: Days After Full Bloom.

**Additional file 3: Fig. S3.** Schematic representation of the development of peach reproductive tissues. Bud dormancy, flowering, and peach fruit developmental processes during the growth season. Only the endodormancy phase is reported for buds, which is overcome after exposure to low temperature. (Chilling Requirement expressed as Chilling Units, CU). The following stages (bud sprouting and blooming) are reached by experimenting with warm temperature. Peach fruit growth follows a double sigmoid kinetic. The double sigmoid curve is the best model for drupe growth in which two exponential growth phases (named S1 and S3) are separated by a slow growth phase (S2), during which the lignification of endocarp occurs. The last phase, named S4, is characterized by fruit ripening. DAFB: days after full bloom; CU: chilling units.

**Additional file 4: Table S1.** Primers RT-PCR and ChIP analyses. Primer sequences used for expression and ChIP analyses. Oligonucleotides were designed, for expression RT-PCR, on primary transcript corresponding to target gene sequences, including the housekeeping gene. For the ChIP investigation, the relative position to predict genomic TSS (from http://phytozome.jgi.doe.gov) is reported for each oligonucleotide. All the sequences are reported in a 5ʹ-3ʹ orientation.

**Additional file 5.** Supplemental references.

## Data Availability

RNA-Seq and ChIP-Seq data from this article can be found in the Gene Expression Omnibus data library under Accession number GSE189882 (https://www.ncbi.nlm.nih.gov/geo/query/acc.cgi?acc=GSE189882), and GSE190586 (https://www.ncbi.nlm.nih.gov/geo/query/acc.cgi?acc=GSE190586), respectively.
