# Supplementary figures and images for: An efficient chromatin immunoprecipitation (ChIP) protocol for studying histone modifications in peach reproductive tissues
Source: Plant Methods. 2022 Mar 31;18:43. doi: 10.1186/s13007-022-00876-0 (PMC8973749; doi:10.1186/s13007-022-00876-0)

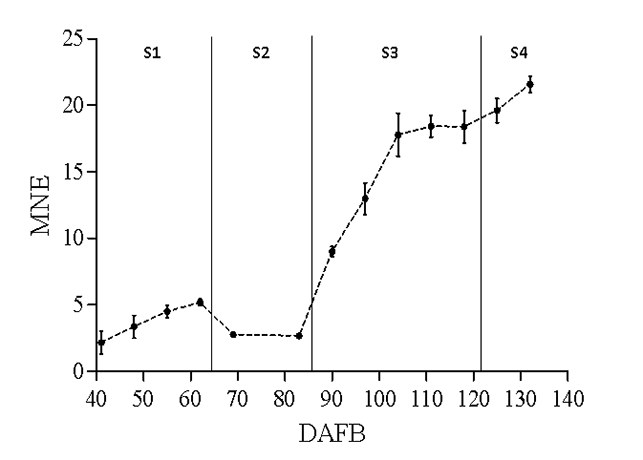

Supplement: Supplementary file 1 — Additional file 1: Fig. S1. Expression pattern of Fleshy (PRUPE_6G159200) gene throughout the whole fruit development in Fantasia genotype. Quantitative real-time qRT-PCRs were performed throughout fruit development in the mesocarp of cv FAN (for details and primers sequences see “Material and methods” section and Additional file 4: Table S1). Developmental phases S1, S2, S3, and S4, related to the typical growth kinetic of the peach fruit, are indicated at the top of each chart. The analysis shows a differential expression pattern during fruit development, displaying an exponential increase in expression levels starting from the end of S2 up to the S4 stage. qRT-PCRs were performed in triplicate on three biological replicates as described by [1] and [2]. Data were acquired, elaborated, and exported with the StepOne Software v2.3 (ThermoFisher, Waltham, MA, USA) and the average value was graphed. MNE: Mean Normalized Expression, DAFB: days after full bloom. Bars represent standard deviation (n = 3). [file 13007_2022_876_MOESM1_ESM.tif]

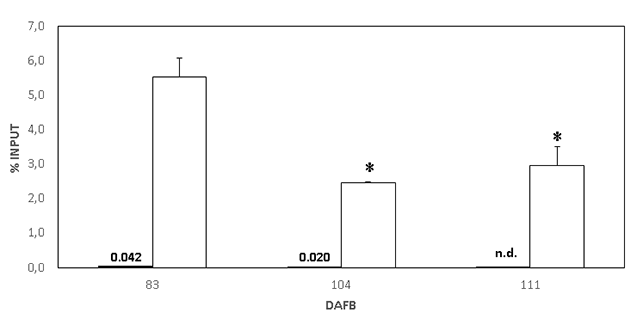

Supplement: Supplementary file 2 — Additional file 2: Fig. S2. Histone modification analysis on gene body-ppePG22 genomic locus. Chromatin marks analysis by the X-ChIP method was performed on chromatin extracted from FAN mesocarp tissue at 83, 104, and 111 DAFB. The ‘gene body-’ (questioned with two pairs of primer set designed on CDS) were investigated by real-time PCR quantification on ChIPed DNA immunoprecipitated with α-H3K4me3 (black bars, not visible) and α-H3K27me3 (white bars). Data are reported as a percentage of chromatin input (% INPUT), normalized on background signal (No Ab serum control sample, measured by omitting antibody during ChIP procedure). Three PCR repetitions for each ChIP assay. Standard errors are reported. Asterisks indicate statistically significant changes of * = P ˂ 0.05. DAFB: Days After Full Bloom. [file 13007_2022_876_MOESM2_ESM.tif]

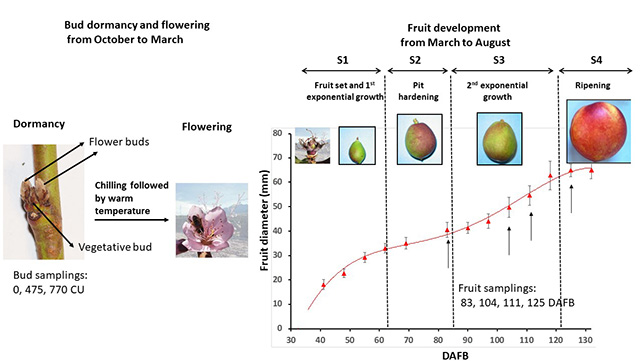

Supplement: Supplementary file 3 — Additional file 3: Fig. S3. Schematic representation of the development of peach reproductive tissues. Bud dormancy, flowering, and peach fruit developmental processes during the growth season. Only the endodormancy phase is reported for buds, which is overcome after exposure to low temperature. (Chilling Requirement expressed as Chilling Units, CU). The following stages (bud sprouting and blooming) are reached by experimenting with warm temperature. Peach fruit growth follows a double sigmoid kinetic. The double sigmoid curve is the best model for drupe growth in which two exponential growth phases (named S1 and S3) are separated by a slow growth phase (S2), during which the lignification of endocarp occurs. The last phase, named S4, is characterized by fruit ripening. DAFB: days after full bloom; CU: chilling units. [file 13007_2022_876_MOESM3_ESM.jpg]
