# Supplementary material for: An efficient chromatin immunoprecipitation (ChIP) protocol for studying histone modifications in peach reproductive tissues
Source: Plant Methods. 2022 Mar 31;18:43. doi: 10.1186/s13007-022-00876-0 (PMC8973749; doi:10.1186/s13007-022-00876-0)
Supplement: Supplementary file 4 — Additional file 4: Table S1. Primers RT-PCR and ChIP analyses. Primer sequences used for expression and ChIP analyses. Oligonucleotides were designed, for expression RT-PCR, on primary transcript corresponding to target gene sequences, including the housekeeping gene. For the ChIP investigation, the relative position to predict genomic TSS (from http://phytozome.jgi.doe.gov) is reported for each oligonucleotide. All the sequences are reported in a 5ʹ-3ʹ orientation. [file 13007_2022_876_MOESM4_ESM.pdf]

Expression analysis_qRT-PCR			ChIP analysis_qPCR
P. persica ID		**Primer sequence 5'>3'**	**Primer sequence 5'>3' and relativeposition to predict TSS**
**PRUPE_4G204900**	**ppeUBQ**	Fw: AAGGCTAAGATCCAAGACAAAGAG<br>Rev: CCACGAAGACGAAGCACTAAG	
**PRUPE_6G159200**	**ppeFLYind**	Fw: GTGCGAATCAGTGACGACCCG<br>Rev: GTCTTTGGAGGATGCGGATTTTCTC	FwA: CGGTTACTTTGGAATGTATTGTGAG (promoter)<br>RevA: AACGAAACGGAGTGTAAGTGGAG (5'UTR)
			FwB: AAAGAAGCAGCAGCGCCAGG (5'UTR)<br>RevB: ATTGGGTGTCAGGTGGGGCC (5'UTR)
			FwC: CGAATATTCTTCTCTTTGGCC (CDS)<br>RevC: TCACCACCACCACCATCACCT (CDS)
			FwD: AGGTGATGGTGGTGGTGGTGA (CDS)<br>RevD: GTCTTTGGAGGATGCGGATTTTCTC (CDS)
**PRUPE_4G262200**	**ppePG22**		Fw1: TTGTCCCAGCGGAGCAACGG (CDS)<br>Rev1: ATTTGATTACGTTCTGTGGAGTTTG (CDS)<br>Fw2: CTGGTCAGGTAAGATATCATTCATGC (CDS)<br>Rev2: GAGCAAGCCCATGGAGGAAGG (CDS)
