# Supplementary material for: An efficient chromatin immunoprecipitation (ChIP) protocol for studying histone modifications in peach reproductive tissues
Source: Plant Methods. 2022 Mar 31;18:43. doi: 10.1186/s13007-022-00876-0 (PMC8973749; doi:10.1186/s13007-022-00876-0)
Supplement: Supplementary file 5 — Additional file 5. Supplemental references. [file 13007_2022_876_MOESM5_ESM.docx]

**ADDITIONAL REFERENCES**

[1] Ziliotto F, Corso M, Rizzini FM, Rasori A, Botton A, Bonghi C. Grape berry ripening delay induced by a pre-véraison NAA treatment is paralleled by a shift in the expression pattern of auxin- and ethylene-related genes. BMC Plant Biol 2012;12. https://doi.org/10.1186/1471-2229-12-185.

[2] Eccher G, Botton A, Dimauro M, Boschetti A, Ruperti B, Ramina A. Early induction of apple fruitlet abscission is characterized by an increase of both isoprene emission and abscisic acid content. Plant Physiol 2013;161:1952–69. https://doi.org/10.1104/pp.112.208470.
